# Lipemia retinalis secondary to pituitary neuroendocrine tumor: a case report

**DOI:** 10.3389/fmed.2026.1888102

**Published:** 2026-08-05

**Authors:** Tingting Cui, Lina Meng

**Affiliations:** Department of Ophthalmology, Qilu Hospital (Qingdao), Cheeloo College of Medicine, Shandong University, Qingdao, China

**Keywords:** chylous blood, growth hormone, hypertriglyceridemia, lipemia retinalis, pituitary neuroendocrine tumor

## Abstract

This case report presents a 31-year-old male patient with lipemia retinalis secondary to a pituitary neuroendocrine tumor (PitNET). The patient presented with a 3-year history of progressive facial changes and enlargement of the hands and feet. Physical examination revealed typical signs of acromegaly. Cranial magnetic resonance imaging (MRI) confirmed a pituitary tumor. Laboratory examinations revealed significant elevations in growth hormone, insulin-like growth factor-1 (IGF-1), and serum lipid levels, along with chylous blood. Bilateral fundus examination showed characteristic creamy-pink discoloration of retinal arteries and veins, as well as a darkened retina with slight salmon-pink discoloration. After treatment with lipid-lowering therapy, glycemic control, and pituitary tumor resection, the patient’s serum lipid levels, blood glucose, and growth hormone levels returned to normal, and the fundus manifestations resolved completely. Lipemia retinalis is an uncommon ocular disorder, and its occurrence secondary to a pituitary tumor is extremely rare. Clinicians are advised to closely monitor lipid metabolism and ocular manifestations in patients with pituitary tumors. Early identification of lipemia retinalis can help prevent serious adverse outcomes such as retinal vascular occlusion and systemic vascular complications.

## Introduction

The pituitary neuroendocrine tumor (PitNET) presented in this case report secreted excessive growth hormone. Persistent hormonal dysregulation may trigger multiple complications, including acromegaly and various metabolic disorders. However, lipemia retinalis secondary to PitNET is extremely rare. This case report presents a 31-year-old male patient with lipemia retinalis secondary to PitNET and aims to provide a reference for the clinical diagnosis and treatment of similar cases.

## Case description

**Figure 1:**
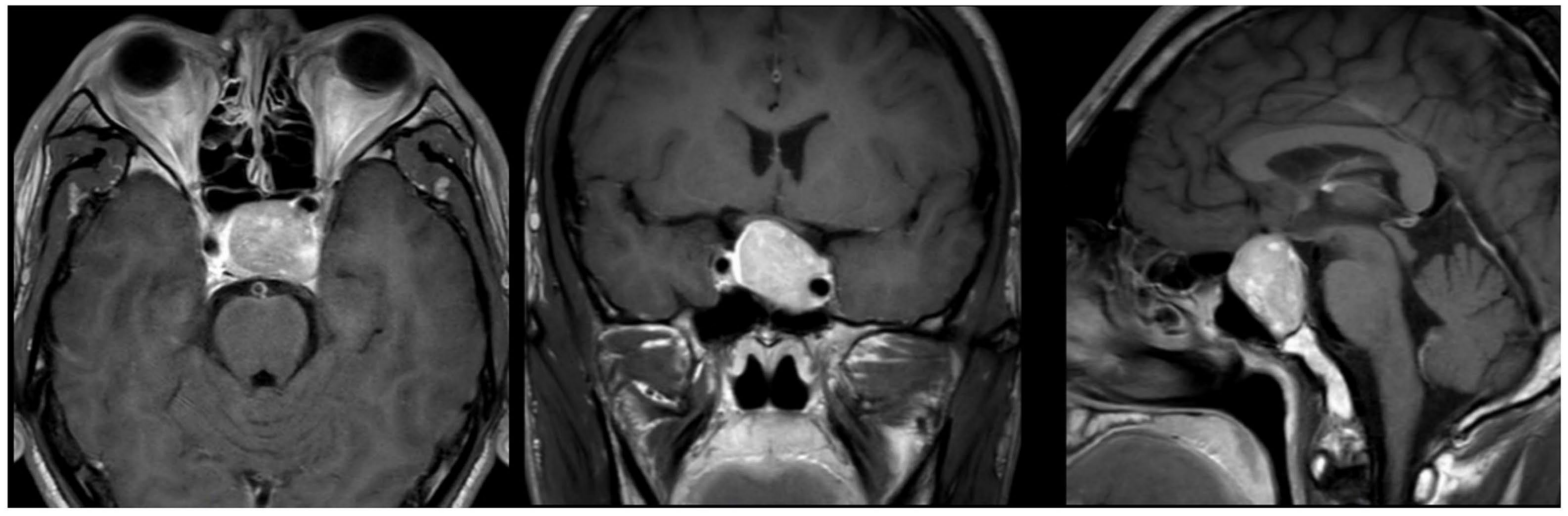
Cranial magnetic resonance imaging (MRI) reveals a pituitary tumor measuring 2.9 × 2.6 × 3.3 cm.

A 31-year-old man presented with a 3-year history of progressive facial changes and enlargement of the hands and feet, accompanied by long-standing, poorly managed diabetes. His height was 182 cm and body weight was 85 kg. Physical examination showed stable vital signs, clear mentation, and a good general condition. Typical acromegalic manifestations were noted, including protruding supraorbital ridges, hypertrophy of the nasal alae, thickened lips, enlarged hands and feet, and coarse skin. Cranial magnetic resonance imaging (MRI) revealed a pituitary tumor measuring 2.9 × 2.6 × 3.3 cm (Figure 1). The pituitary stalk was compressed and deviated to the right. The optic nerves were compressed and displaced. The bilateral cavernous segments of the internal carotid arteries were compressed and displaced, with partial encasement of the left internal carotid artery. The tumor was classified as Knosp grade 3. Laboratory examinations demonstrated a fasting blood glucose (FBG) level of 18.47 mmol/L and a glycosylated hemoglobin level of 12.13%. Serum growth hormone (GH) was above 50 ng/mL (reference range: 0–2.47 ng/mL), and insulin-like growth factor-1 (IGF-1) reached 663.98 ng/mL (reference range: 138.4–263.3 ng/mL). Serum total cholesterol was 18.62 mmol/L (reference range: 2.8–6.0 mmol/L), while the triglyceride level reached 37.42 mmol/L (reference range: 0.3–1.7 mmol/L). A chylous appearance was observed in peripheral blood samples. Serum luteinizing hormone (LH) was 4.39 mIU/mL, prolactin (PRL) was 92.56 mIU/L, and thyroid-stimulating hormone (TSH) was 0.478 μIU/mL; all values were within the reference ranges. No remarkable abnormalities were detected in routine blood tests, hepatic or renal function, serum electrolyte levels, or amylase levels.

**Figure 2:**
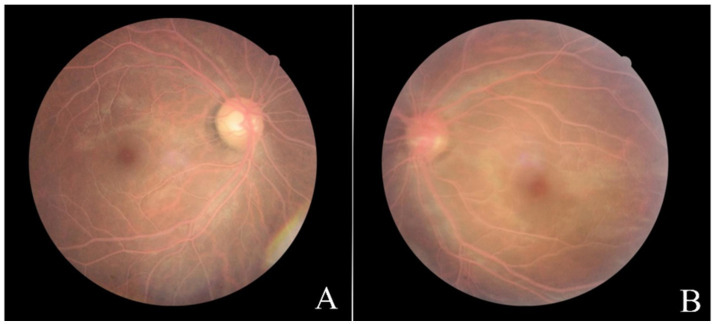
Fundus photography shows creamy-pink discoloration of retinal arteries and veins, as well as darkening of the retina with slight salmon-pink changes (panel **A** shows the right eye, and panel **B** shows the left eye).

The patient had a history of congenital amblyopia in the left eye, with no evident visual deterioration over the past 3 years. Ocular examinations revealed the following results: best-corrected visual acuity (BCVA) was 1.0 (−0.50 DS) in the right eye and 0.02 (+2.00 DS/+1.50 DC × 95°) in the left eye. Both corneas were transparent; the anterior chamber fluid was clear; the pupils were round; and the crystalline lenses remained clear. No palpebral xanthelasma was observed in either eye. Fundus photography showed a creamy-pink discoloration of the retinal arteries and veins, as well as darkening of the retina with slight salmon-pink changes (Figure 2), consistent with the typical features of lipemia retinalis. No retinal hemorrhage or exudation was observed in either eye. Bilateral visual field testing revealed no significant quadrantic field defects.

During hospitalization, the patient received oral atorvastatin for lipid-lowering therapy and insulin to control blood glucose levels. The patient underwent endoscopic transnasal transsphenoidal pituitary adenomectomy with tumor resection, and the operation proceeded uneventfully. Pathological examination confirmed a pituitary neuroendocrine tumor (pituitary adenoma). The total volume of submitted tissue measured 1.5 × 1.2 × 0.3 cm. Immunohistochemical staining showed that the tumor predominantly expressed PIT-1, with focal SF-1 expression; a plurihormonal tumor was favored. The immunohistochemistry panel showed the following results: PIT-1 (+), SF-1 (focal +), LH (+), GH (weak +), TSH (focal +), PRL (focal +), ACTH (−), FSH (−), T-PIT (−), Syn (+), CgA (+), CD56 (+), GFAP (focal +), GATA-3 (−), CD45 (LCA) (−), CAM5.2 (−), TTF-1 (−), ER (−), and Ki-67 (+, approximately 1%). Given the markedly elevated serum growth hormone level, normal levels of serum LH, PRL, and TSH, together with the associated clinical manifestations, this PitNET was a predominantly growth hormone-secreting tumor. The serum GH level on postoperative day 1 was 5.93 ng/mL, representing a significant decrease from the preoperative baseline. The patient had an uncomplicated postoperative recovery with no adverse events, including fever, headache, or cerebrospinal fluid leakage, and was discharged on postoperative day 4.

**Figure 3:**
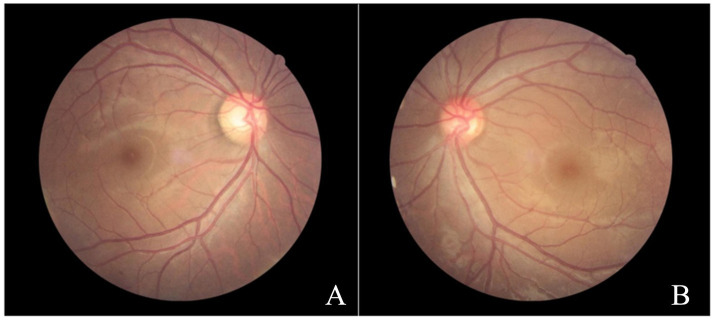
Retinal color returns to normal, and the bilateral retinal arteries and veins restore their normal reddish appearance (panel **A** shows the right eye, and panel **B** shows the left eye).

Thirteen days postoperatively, outpatient follow-up revealed notable improvements in laboratory indicators: total cholesterol decreased to 2.35 mmol/L, triglycerides to 1.04 mmol/L, and growth hormone to 2.03 ng/mL, all of which fell within their normal reference ranges, and atorvastatin was discontinued thereafter. The serum IGF-1 level decreased to 440.47 ng/mL, although it had not returned to the normal reference range. However, FBG remained persistently elevated at 17.96 mmol/L, necessitating further optimization of insulin treatment regimens. Postoperative fundus examination showed that retinal color had returned to normal, and the bilateral retinal arteries and veins had been restored to their normal reddish appearance (Figure 3). On postoperative day 41, the serum IGF-1 level decreased to 424.88 ng/mL. FBG was 8.69 mmol/L, representing a marked improvement compared with previous measurements. Total cholesterol was 5.02 mmol/L, and triglycerides were 1.50 mmol/L, both within normal limits. The patient was instructed to attend regular follow-ups at the endocrinology department, adjust insulin doses to achieve satisfactory glycemic control, and undergo periodic monitoring of blood glucose, serum lipids, growth hormone, and IGF-1, as well as periodic cranial MRI examinations.

At the 3-month postoperative follow-up, the patient maintained stable glycemic control. Neither recurrent fundus abnormalities nor progressive acromegalic manifestations were noted. Regrettably, the patient did not undergo a follow-up IGF-1 measurement. Biochemical remission could not be confirmed in this patient. He was satisfied with the treatment.

## Discussion

Lipemia retinalis is an uncommon ocular sign secondary to severe hypertriglyceridemia, characterized by a creamy-white discoloration of the retinal blood vessels. It can be triggered by various causes, such as primary hyperlipidemia, hyperlipidemia caused by poorly controlled diabetes, lipid metabolic disorders, alcohol abuse, or medications (1–4). It is a rare retinal manifestation that typically occurs at serum triglyceride levels above 28.25 mmol/L (2,500 mg/dL) (5–7). The incidence of lipemia retinalis correlates approximately with plasma triglyceride levels. However, the relationship is indirect because the key factor is the level of chylomicrons, the large lipoproteins that transport triglycerides. The fundus appearance is believed to result from the visualization of increased levels of chylomicrons in the circulating retinal blood. However, not all patients with even grossly elevated levels of chylomicrons or triglycerides show evidence of lipemia retinalis. Other factors, such as hematocrit and differences in the translucency of the retinal and choroidal vessels, are important (8). The principal fundus abnormality in lipemia retinalis is a milky discoloration of the retinal vessels. This can vary in severity from barely detectable peripheral vessel changes to a cream coloration of all retinal vessels with a salmon-colored fundus (1).

In the present case, the patient had a serum triglyceride level of 37.42 mmol/L with obvious chylous blood. Fundus photography showed creamy-pink discoloration of the retinal arteries and veins, as well as darkening of the retina with slight salmon-pink changes in both eyes, consistent with the typical features of lipemia retinalis. Lipemia retinalis in this patient was caused by excessive growth hormone secretion from the PitNET. GH-induced signaling activates the MEK–ERK pathway, which results in FSP27 downregulation. The decrease in FSP27 expression leads to increased lipolysis and higher levels of circulating free fatty acids (FFAs). In addition, GH induces lipolysis by activating hormone-sensitive lipase (HSL) and by increasing *de novo* expression of HSL mRNA via the activation of protein kinase C and ERK. GH suppresses the activity of lipoprotein lipase and inhibits FFA uptake into adipose tissue (9, 10). Elevated circulating FFAs directly stimulate hepatic triglyceride synthesis, thereby increasing plasma triglyceride (TG) levels. In summary, excessively elevated growth hormone mainly affects lipid metabolism by promoting lipolysis and increasing free fatty acid levels, which subsequently leads to hypertriglyceridemia and ultimately contributes to the development of lipemia retinalis. In addition, the patient had long-standing, poorly controlled diabetes mellitus, which may aggravate lipid metabolism disorders and further exacerbate lipemia retinalis. Given the patient’s long-standing suboptimal glycemic control, underlying risk of systemic atherosclerosis, and concurrent hyperlipidemia, atorvastatin was administered to mitigate cardiovascular risks. After pituitary tumor resection, both growth hormone and triglyceride levels returned to normal, accompanied by complete resolution of the fundus abnormalities. The underlying cause of hyperlipidemia in this patient was excessive growth hormone secretion. Excessively elevated growth hormone constituted a major pathogenic factor leading to a severe lipid metabolism disorder. Adenomectomy served as the definitive curative intervention, and its therapeutic effect far outweighed that of lipid-lowering medications.

Patients with PitNETs that oversecrete growth hormone should receive routine lipid profiling and ophthalmologic evaluation to avoid missing a diagnosis of rare complications such as lipemia retinalis. Therapeutically, the core principle is to eliminate the underlying cause, namely surgical resection of the PitNET to suppress excessive growth hormone secretion, combined with active management of metabolic disorders including glycemic control and lipid-lowering interventions. The IGF-1 level continued to decrease at 41 days postoperatively, yet the magnitude of decline was modest compared with the value measured on postoperative day 13. Somatostatin analogs may be considered if IGF-1 remains elevated during follow-up examination. As for lipemia retinalis, fundus abnormalities generally resolve gradually as serum lipid levels return to normal, and no specific targeted ocular therapy is required. Lipemia retinalis is usually asymptomatic and is often detected incidentally. The patient’s BCVA in the left eye was only 0.02. This severe visual loss was attributable to a pre-existing history of congenital amblyopia and was unrelated to the current lipemia retinalis. Although fundus lesions are reversible, severe hypertriglyceridemia during the active stage of the disease can lead to serious systemic complications. Chronically elevated triglycerides accelerate systemic atherosclerosis and damage vascular endothelium in both systemic and ocular circulations, subsequently triggering severe vascular complications, including retinal vascular occlusion (11). Severe hypertriglyceridemia can also precipitate acute pancreatitis (12), which is characterized by sudden onset and rapid progression, with severe cases being associated with high mortality. Therefore, clinicians should maintain close clinical vigilance and perform early identification and timely intervention to prevent these severe complications. This approach is of great clinical value for improving patients’ overall prognosis and reducing disease-related mortality and disability rates.

This case report has several limitations. The patient did not attend the scheduled IGF-1 follow-up examination 3 months after surgery; therefore, biochemical remission of acromegaly could not be confirmed. Given that this was a Knosp grade 3 tumor, only partial tumor tissue was collected for pathological examination intraoperatively to protect the optic nerves and internal carotid arteries. The residual tumor tissue was suctioned and was not included in the pathological specimens, resulting in a smaller pathological specimen volume than the pituitary tumor volume measured on preoperative MRI. Therefore, the presence of minimal residual tumor cannot be ruled out. Postoperative MRI was not performed, making it impossible to evaluate for residual or recurrent pituitary tumor.

Very few reports have described lipemia retinalis secondary to PitNET. This case report provides a practical reference for the clinical diagnosis and treatment of this rare condition. It also alerts clinicians to closely monitor patients’ lipid metabolism and ocular manifestations during the management of PitNET with excessive growth hormone secretion.

## Data Availability

The original contributions presented in the study are included in the article/supplementary material, further inquiries can be directed to the corresponding author.
